# Relationship between three palliative care outcome scales

**DOI:** 10.1186/1477-7525-2-68

**Published:** 2004-11-29

**Authors:** Irene J Higginson, Nora Donaldson

**Affiliations:** 1 Department of Palliative Care and Policy, King's College London, Weston Education Centre, Cutcombe Road, London SE5 9RJ, UK

**Keywords:** palliative care, quality of life, assessment, hope, symptoms, hospice, day care

## Abstract

**Background:**

Various scales have been used to assess palliative outcomes. But measurement can still be problematic and core components of measures have not been identified. This study aimed to determine the relationships between, and factorial structure of, three widely used scales among advanced cancer patients.

**Methods:**

Patients were recruited who received home or hospital palliative care services in the south of England. Hope, quality of life and palliative outcomes were assessed by patients in face to face interviews, using three previously established scales – a generic measure (EQoL), a palliative care specific measure (POS) and a measure of hope (Herth Hope Index). Analysis comprised: exploratory factor analysis of each individual scale, and all scales combined, and confirmatory factor analysis for model building and validation.

**Results:**

Of 171 patients identified, 140 (81%) consented and completed first interviews; mean age was 71 years, 54% were women, 132 had cancer. In exploratory analysis of individual means, three out of the five factors in the EQoL explained 75% of its variability, four out of the 10 factors in POS explained 63% of its variability, and in the Hope Index, nine out of the 12 items explained 69% of its variability. When exploring the relative factorial structure of all three scales, five factors explained 56% of total combined variability. Confirmatory analysis reduced this to a model with four factors – self-sufficiency, positivity, symptoms and spiritual. Removal of the spiritual factor left a model with an improved goodness of fit and a measure with 11 items.

**Conclusion:**

We identified three factors which are important outcomes and would be simple to measure in clinical practice and research.

## Background

Measurement of the effect of illness and its treatment on patients is now an accepted part of clinical trial design [1]. Such measurement is also proposed as an aid to improve clinical practice and decision making [2,3]. However, as the illness becomes more advanced the value of many well validated quality of life instruments has been challenged [4-9]. There are three main difficulties. First, many quality of life scales focus on the assessment of physical functioning, which deteriorates as illness progresses [4,8]. This can render the measure insensitive to, or mask, other changes. Second, most quality of life scales have been validated among patients in early stage illness, such as cancer or whilst undergoing chemotherapy or curative treatment [8,9]. Sometimes their validation was founded on an assumption that patients in terminal disease had a poorer quality of life than those at diagnosis [10]. This assumption has been consistently challenged [8]. Concerns among patients with more advanced illness are often different to earlier stages, as patients reframe their priorities in the face of impending death [8]. Existential, relationships, information, the provision of care, and use of remaining time become more important [9]. Third, collecting information from patients at late stages of disease is practically difficult; questionnaires need to be kept short, be easy to use, and be few in number. Even then there are often difficulties of missing data and loss to follow-up [8,9].

In response to these difficulties, different measures have been developed and tested among patients receiving palliative and hospice care in different countries and contexts [8,11]. These include scales that assess, to different degrees, symptoms, existential aspects or spirituality, the impact of therapy, hope, information, social and family concerns [8,9,12]. Some are completed directly by patients, some by family members or other proxies, and some by a combination of these. However, there is little information on how different measures compare, particularly in relation to more traditional measures. Clinicians and researchers need such information to determine which core factors should be measured, especially when it is not possible to collect a battery of measures. This study therefore sought to determine the relationships between three such scales and their factorial structures to recommend short, self-contained scales for future use among people with advanced cancer.

## Methods

### Design

Secondary analysis of a prospective observational study.

### Patients and setting

Patients living in Chichester in the South of England receiving home or hospital palliative care support, from community, hospice or hospital palliative care team staff, were approached to take part in the study. Local research ethics committee approval was obtained. The local hospice was planning to develop a day care unit and patients were recruited during this period. A historical group was recruited before the day care unit was established. Consecutive consenting patients were recruited for both series. Patients were eligible if they were in the care of the hospice home care team, or neighbouring home care teams, that had access to the day care unit. Patients were excluded if they were judged by staff to be too ill for interview, if staff felt it would distress them, or they lived outside the catchment area of the hospice day unit. Two concurrent groups were recruited after day care was established – patients who did (Group AD) and did not (Group AN) choose to receive day care.

### Data collection

Data was collected using trained interviewers. Interviews took place in the patients' preferred location, usually their own home. Interviewers explained the background to the study and used a structured schedule to collect clinical, demographic and use of service data. They then administered three scales. All were short, taking less than 10 minutes on average to complete, and were acceptable to advanced cancer patients. Scales were administered in the order they are listed below.

1. EQoL EQ-5D. This generic questionnaire defines health in five dimensions: mobility, self-care, usual activities, pain or discomfort, and anxiety or depression. Each dimension is divided into three categories – whether the respondent has no problem, a moderate problem, or an extreme problem. A sixth item scores the person's overall health on a visual analogue (0 – 100) scale. The questionnaire has been validated and applied in a wide range of patient groups [13-16].

2. Palliative care Outcome Scale (POS). This 10 item scale (plus an open question) was specifically developed and validated for palliative care and covers physical symptoms, patient and family or caregiver anxiety/fears and well being. The effect of the items on the daily life of the patient over the past three days is scored on a five-point Likert scale ranging from 'none' (0) to 'overwhelmingly' (4). For example: "over the past 3 days, have you been feeling anxious or worried about your illness or treatment? (0) not at all – (4) overwhelmingly" [17,18]. In the POS the term 'family' describes the caregiver or significant other, such as a partner, spouse or other closest individual.

3. Herth Hope Index (Hope). This 12 item instrument assesses hope in adults in clinical settings, and is designed to assess change. For example: "I have a positive outlook toward life? strongly disagree (1) to strongly agree (4) -". Patients are asked how much they agree with the statement right now [19].

Full details of the scales are shown in the Appendix 1 (see additional file 1). Patients were interviewed immediately after referral to the study. Follow-up interviews occurred but these data are not considered here.

### Analysis

Data were analysed separately for the historical and concurrent (post day care) groups. The relative factorial structure of the three scales was explored in two steps. First, we performed a preliminary exploratory factor analysis (EFA) on each individual scale and on all the items of the three scales combined, using Principal Component Analysis on the historical sample. Second, we performed further exploration and final validation using confirmatory factor analysis (CFA) on the combined historical and concurrent samples. The EQS software [20] was used to compare several models to the covariance matrix of the 28 variables. Although this was an observational follow-up study, for the purpose of this paper we always used the baseline measures, when complete data for all patients was available.

## Results

171 patients were identified and asked to take part in the study, 82 in the historical group, and 89 in the concurrent group (40 were AD). Of these, 140 (81%) were successfully approached, agreed to take part in the study, and completed the first interview. As shown in Table 1, 66 were from the historical group and 74 were from the concurrent group (of whom 28 were AD). Failure to interview was due to: refused 12, felt too unwell 11, died 8. Complete data in all three scales were obtained in 137 patients. As Table 1 shows the AD and AN were similar, and so were subsequently merged to form the concurrent group. The concurrent and historical samples were very similar in terms of characteristics like age, ethnicity, willingness to take part in the study, diagnosis, as well as their relationship to the carer and whether they resided with family, spouse or alone and housing. In spite of the age similarity the proportion of retired people was slightly larger in the concurrent sample. Differences between the two samples were only observed for place of death and for gender. Although not statistically significant, the concurrent sample tended to have more patients dying at home while the historical sample tended to have more patients dying in hospice. The proportion of women was larger in the historical sample (60% vs 40% P = 0.02). The distribution of cancers was similar to those in the general population.

**Table 1:** Patient socio-demographics (completed 1st interview) for historical group and concurrent group who did (AD) and did not (AN) receive day care

**Demographics**	**Historical Group (n = 66)**	**Group AD (n = 28)**	**Group AN (n = 46)**
**Age in years**			
Mean (SD)	69.2 (12.4)	74.0 (10.1)	70.8 (11.9)
Median/range	71.0/34–94	77.0/50–94	72.0/39–90
**Gender**			
Women	40 (61%)	12 (43%)	23 (50%)
Men	26 (39%)	16 (57%)	23 (50%)
**Ethnicity**			
White UK	66 (100%)	28 (100%)	46 (100%)
**Employment Status**			
Working (F/T or P/T)	6 (9%)	2 (7%)	2 (4.5%)
Not working (unable)	14 (22%)	2 (7%)	9 (20.5%)
Retired	45 (69%)	23 (85%)	33 (75%)
**Carer**			
Spouse	43 (65%)	20 (71%)	30 (70%)
Other carer	12 (18%)	5 (18%)	9 (21%)
No carer	11 (17%)	3 (11%)	4 (9%)
**Carer Contact**			
Lives with spouse	43 (65%)	20 (71%)	30 (70%)
Lives with family	2 (3%)	0	2 (5%)
Lives alone	21 (32%)	8 (29%)	11(25%)
**Carer employment**			
Working (F/T or P/T)	18 (29%)	6 (21%)	8 (19%)
Not working (unable)	5 (8%)	2 (7%)	4 (9%)
Retired	29 (46%)	17 (61%)	27 (63%)
No carer	11 (17%)	3 (11%)	4 (9%)
**Housing**			
Own/private	28 (42%)	11 (39%)	17 (37%)
Own/council	7 (11%)	7 (25%)	7 (15%)
Own/rented	28 (42%)	9 (32%)	20 (44%)
Other (N/home)	3 (5%)	1 (4%)	2 (4%)
**Primary diagnosis**			
Lung cancer	11 (17%)	4 (14%)	11 (26%)
Gastrointestinal	11 (17%)	8 (29%)	9 (21%)
Breast	9 (14%)	4 (14%)	4 (10%)
GU/Prostate	11 (17%)	6 (21%)	8 (19%)
Gynae	7 (11%)	0	3 (7%)
Other cancer	10 (15%)	4 (14%)	7 (17%)
Non-cancer	6 (9%)	2 (7%)	0
**Place of death**	**(n = 46)**	**(n = 11)**	**(n = 22)**
Home	8 (17%)	4 (36%)	6 (27%)
Hospital	7 (15%)	1 (9%)	3 (14%)
Hospice	31 (67%)	6 (55%)	13 (59%)

### Individual Scales

Summaries of the distribution of scores on the three instruments assessing hope, quality of life and palliative outcomes for the combined sample as well as results of the exploratory factor analysis, are shown in Table 4 (see additional file 2). On principal component analysis (unrotated), three factors in EQoL explained 75% of the total variability brought up by the six items in this scale. The first factor, explaining 40%, comprised general health: Health Status and the three self-sufficiency items. Anxiety-Depression defined the second factor, which explained 20% of the variability, and Pain-Discomfort formed the third factor, which explained 15% of the variability. For POS, the exploratory factor analysis grouped the 10 POS items in four factors explaining 67% of its variability. The first factor, which alone explained 27% of the variability, consisted of the two items measuring positivism (life-worthwhile and feel-good) and in addition, worry-anxiety. The second factor, which alone explained 16% of the variability, was mainly determined by information, followed by time-wasted and practical-matters. The third factor, which explained 12% of the variability, was solely determined by the item family-anxious. The fourth factor, also explaining 12% of the variability, was determined by pain and symptom-control. In the individual exploration we found that the 12 items of Hope grouped into four factors that explained approximately 69% of the variability present in the scale. The first factor was items 1, 8, 10, 11, and 12 representing positivity (39%), the second factor had items 2 and 4, measures of goals (12%), the third was items 3 and 6 (10%) and the fourth was items 7, 9 and 5 (9%). These last two factors represented a measure of support loneliness.

### Three scales combined

The exploratoty factor analysis of EQoL, POS and Hope on the historical sample alone gave rather consistent results for different extraction methods. Table 4 (see additional file 2) shows the results of the unrotated principal component analysis. Five factors explained 54% of the total variability present in the three combined scales. The **first **principal factor, explaining 25% of the total variability of the combined scales, was determined by the three items of positivity contained in POS (share-feelings, feel-good and life worthwhile), together with all the Hope items and the anxiety items in both, POS and EQoL. The EQoL items "General Health" and items of "self-sufficiency", which constituted the most important factor of the EQoL scale, loaded together into the **second **factor, explaining 10% of the total variability of the combined scale. The **third **most important principal component, explaining 8% of the variability, comprised a general measure of patient anxiety (measured by both EQoL and POS), and family anxiety (measured by POS). The fourth principal component explained 6% of the variability and was defined by pain (measured by both EQoL and POS). The POS items "information" and "time-wasted" loaded together into the fifth factor, explaining only 6% of the total variability. In addition, the POS item "symptom control" did not load into any of these five factors and appeared to be acting independently.

One of the extractions explored was principal axis factoring with a varimax rotation. This provided a better definition of the structure, with items loading more exclusively onto one of the factors. The first factor that we had obtained with the unrotated matrix essentially separated into two. The first axis, explaining 29% of the variability, was defined by the POS item "life worthwhile" loading with those items of Hope that reflected positivity and direction: positive outlook, goals, inner strength, loving, sense of direction, days have potential and life has value. The second axis, explained 11% of the variability and contained the anxiety items of EQoL and POS, the "feel good" and "share feeling" items of POS and the items of Hope that reflected pessimism or anxiety: "alone", "scared of future" and "past memories". The third factor was the EQoL general health and self-sufficiency and explained 7% of the variability. The fourth factor was solely defined by the pain items in EQoL and POS and explained 6% of the variability. The rest of the items played only a minor role. The POS items: practical matters, information and time wasted loading in a minor factor while the POS item "family-anxious" and the Hope items "tunnel" and "faith" disappeared altogether from the rotated matrix. Therefore, the model derived from this data is one in which the following items are omitted: POS2 and POS4 from the POS scale and Hope4 Hope5 from the Hope scale, leaving the rest of the items to define four major latent factors in the following manner: Spiritual, positivity, symptoms and self-sufficiency.

### Confirmatory Factor Analysis (CFA)

Several models were explored and the most relevant are presented in Table 2 with the various measures of fit given by EQS. Model 1 was a three-factor model allowing each scale (EQoL, POS and Hope) to individually determine each factor. The goodness of fit measures suggest that the model does not provide a good fit for the data, although most of the residuals (observed-predicted covariances) were found to be relatively small and their frequency distribution is symmetric and centred around zero [21]. This model confirmed that the POS and Hope factors were indeed very highly correlated [Estimated correlation = 0.81; 95% CI (0.71–0.91)]. Consequently, a second model was fitted to the data in which only two factors were postulated, the first was the EQoL items as in the previous model and the second factor having as its indicators both the POS variables and the HOPE variables. The fit was very similar to the fit of Model 1 but it appeared that the two factor model needs to be considered as a serious alternative to model 1. In addition, the results of these two models suggest that some of the POS variables (family anxious, information given, time wasted and practical matters) are not needed for defining the second factor. As a result of this, we explored a range of models, allowing for the strong correlation between POS and Hope and giving special attention to those items that were unimportant in either the exploratory or preliminary confirmatory factor analysis. Three of the POS items, which confirmed a latent construct that we called "practical", proved to be superfluous in the overall construct. These items were: information given, time wasted and practical matters. The POS item *family-anxious *did not particularly disrupt the identifiability of the model but its presence reduced the goodness of the fit. Similarly, four Hope items were discarded – alone, light at the end of the tunnel, faith and scared of future – to give a total of seven items discarded. We arrived to two models, exhibited in Table 2: Model 3, fitting the four factors listed in Table 3, and Model 4, fitting only the first three factors, leaving out the spiritual factors construed by the Hope scale. Table 2 includes the goodness of fit statistics for these models.

**Table 2:** Goodness of fit summaries for the four models derived by Confirmatory Factor Analysis (CFA)

	Model 1	Model 2	Model 3	Model 4
Independence	1076	1076	717	279
Chi-square	(378 df)	(378 df)	(171)	(55 df)
Average standardised residuals	0.11	0.09	0.09	0.09
Average off-diagonal st. residuals	0.12	0.10	0.10	0.11
Chi-squared fit	534.7	520	213	67.7
(df)s	(347 df)	(347 df)	(150 df)	(43)
P-value	0.00001	0.00001	0.001	0.01
Free parameters	59	57	40	23
Akaike's information criterion (AIC)	-193	-173	-87	-18
Bozodgan's version of AIC (C-AIC)	(-1437)	(-1424)	(-627)	(-173)
Comparative Fit Index (CFI)	0.73	0.75	0.89	0.90
Normed Fit Index (NFI)	0.50	0.52	0.70	0.76
Non-normed Fit Index (NNFI)	0.71	0.73	0.87	0.86

Model 1 comprised the basic 3 factors: EQoL, POS and HOPE. Model 2 was 2 factors: EQoL, and POS and HOPE combined. Model 3 was 4 factors: items relating to self-sufficiency, positivity, symptoms and spiritual. Model 4 was 3 factors, items relating to self-sufficiency, positivity and symptoms.

AIC and CAIC measure degree of fit. The smaller, the better the fit. The larger are NFI, NNI and CFI, the better the fit, with an upper value of 1.

**Table 3:** The factorial structure of the proposed model (MLE Estimators of regression coefficients (Standard Error)

**Scale**	**Item**	**SYMPTOMS**	**SELF SUFFICIENCY**	**POSITIVITY**	**SPIRITUAL**
EQoL1	*Mobility*		**0.32 **(0.06)		
EQoL2	*Self-care*		**0.49 **(0.08)		
EQoL3	*Usual activities*		**0.39 **(0.08)		
EQoL4	*Pain-Discomfort*	**0.38 **(0.08)			
EQoL5	*Anxty-Depression*			**0.34 **(0.06)	
EQoL6	*Health Status*	**-6.9 **(2.34)			
POS1	*Pain Control*	**0.93 **(0.17)			
*POS2*	*Symptom Control*	0.16 (0.11)			
POS3	*Anxious/Worried*			**0.52 **(0.11)	
*POS4*	*Family anxious*			0.26 (0.15)	
*POS5*	*Information*				
POS6	*Share feelings*			**0.69 **(0.15)	
POS7	*Life Worthwhile*			**0.72 **(0.10)	
POS8	*Feel Good*			**0.97 **(0.12)	
*POS9*	*Time Wasted*				
*POS10*	*Practical matters*				
HOP1	*Positive outlook*				**0.42 **(0.07)
*HOP2*	*Goals*				**0.47 **(0.08)
HOP3	*Alone*				
HOP4	*Tunnel*				
HOP5	*Faith*				
HOP6	*Scared of future*				
HOP7	*Happy memories*				**0.27 **(0.07)
*HOP8*	*Inner strength*				**0.65 **(0.08)
HOP9	*Loving*				**0.32 **(0.07)
HOP10	*Sense of direction*				**0.77 **(0.08)
HOP11	*Days Potential*				**0.67 **(0.07)
HOP12	*Life has value*				**0.55 **(0.08)

Significant coefficients are highlighted.

In all the models presented, the matrix was positively definite, the test of independence was significant and the frequency distribution of the standardised residuals was symmetrical around 0. Models 3 and 4, not only omit the superfluous items but also separate the factors on clinical considerations. Both provide a huge improvement over the first two models. Model 3 allowed for a high correlation between the positivity and spiritual factors. More remarkably, the results show that Model 4, which disposes completely of the spiritual factor defined by the remaining Hope items, is an enormous improvement on the other models. The chi-square statistic was greatly reduced and almost reached the threshold indicating that no lack of fit was detected.

## Discussion

An important next step in quality of life measurement is the translation of measurement into clinical practice to improve patient care [2]. One important barrier among patients with advanced illness is ensuring that relevant items are captured from a sufficiently small range of instruments relevant to this stage of illness. The three measures used in this study all have relevance in advanced illness. The EQoL deals with general aspects of health related quality of life, generating within the scale 243 possible health states. It has been used to provide indexed preferences for health states [22], and health state valuations in national and cross cultural studies [23]. Standardised measures, such as the Medical Outcome Study (MOS) short form 12 (SF-12) map to this scale [24]. Among our patients with advanced illness, primarily cancer, we found variability within the EQoL, although patients tended to score at the poorer end of the scale. Health status and the self-sufficiency items of mobility, self-care and usual activities explained 40% of the variation of this scale in our patient population. We included the self-sufficiency aspect in our model of summary factors, but it is debatable whether items such as mobility, self-care and usual activities are relevant outcomes in palliative care. Functional status and those items within quality of life measures that reflect functional status are highly correlated to survival [25], thus the scores will inevitably deteriorate towards death. However, to provide consistency with other scales used in general health care and cancer treatment, measurement may be valuable [24].

A factor which we entitled 'positivity' appeared to be highly important among people with advanced illness. Spirituality/positivity has also been related to communication outcomes [26]. In the exploratory factor analysis it explained 24% of the total variability of the combined scales. Its importance was maintained in the confirmatory factor analysis. In model 3 positivity could be seen as separate from spirituality, but if a smaller model is required, spirituality can be assessed through positivity, because it is strongly correlated. Items that reflect this domain of positivity are found in a number of measures of palliative care [9,12,18]. However, our study is the first to quantify the extent to which this positive domain is relevant in patients with advanced illness. Our data suggests it can be measured in a variety of ways, through questions related to sharing feelings, feeling good, anxiety, as well as questions directly tapping hope.

When attempting to develop a reduced scale we identified two models, one with four factors (19 items) and another one with three factors which provided a good fit (11 items). All the Herth Hope items are excluded from the latter model, which captured self-sufficiency, symptoms and positivity. Positivity appeared very close to spirituality, as measured by the Hope index. Further work is needed to determine the relationship of these questions with specific spirituality scales [27-29].

Symptom control was absent in the structures obtained by EFA. We suspected that this was because this item was not a structured question designed for any specific symptom, but elicited in an open way what symptoms had troubled the patient. In the CFA this item loaded with the General Health status factor. Measures which specifically address symptoms are available and have been used in palliative care populations [29,30]. Work with the POS has now developed to separate symptom modules and these are in the process of further testing and validation [31].

Special attention was given to the three items forming the practical factor in POS. The information item in POS (POS-5) was constant in the concurrent sample and only a few patients in the historical sample recorded non zero for this item. The time wasted item of POS was also essentially constant. It may be that the grading for these items needs to be reviewed to ensure that they give greater sensitivity to change. In our analysis this could have contributed to the poor fit shown when attempting to fit a general POS factor containing these items. It may also be because that none of the three items is an indicator of QOL; they are rather items of the provision of health care. In this study all of the patients were in receipt of a wide range of services, including specialist care teams and their practical needs were likely to have been met. Research among patients in different circumstances has shown deficiencies in these aspects of care [32,33].

The POS item family-anxious was intriguing. It did not disrupt the validity of the model but if kept and loaded in the positivity factor, it reduced the goodness of fit. This item also showed erratic behaviour in the exploratory factor analysis. Family anxiety may be related to a large number of factors, some of which are determined by the circumstances of the patient and some of which are determined by other events. Family needs often increase as patients deteriorate and are difficult to resolve. Further work is required directly targeting the needs of families [34].

Our study was limited by the comparison of these scales among patients in one setting: we do not know if similar results would be found if patients were not in receipt of specialist palliative care available in the United Kingdom. However, our findings are consistent with other work assessing quality of life measurement in palliative care and in advanced cancer [11]. Correlation between the scales may also have occurred because individuals were aware of the answers they had given for the different scales. It would be impossible to avoid this process in the completion of the questionnaires. We did not vary the order in which the questionnaires were administered. However, we believed that the questions appeared to be sufficiently different for patients not to be influenced by their prior responses. Future research should analyse this.

Our reduced model suggests that clinicians may sensibly target quality of life measurement in advanced cancer towards three main components, positivity, self-sufficiency, and symptoms. This might be achieved by the 12 items in model 4 of our factor analysis. Such a scale would be short and simple to use in both clinical practice and research, improving the measurement of outcomes in this population.

## Supplementary Material

Additional File 1 Appendix 1: Full details of the three palliative care outcome scales Click here for file

Additional File 2 Table 4: Summary Statistics and Principal Component Analysis (unrotated) of the three scales (POS-EQoL-Hope) in the historical sample. Click here for file
