# Supplementary material for: Relationship between three palliative care outcome scales
Source: Health Qual Life Outcomes. 2004 Nov 29;2:68. doi: 10.1186/1477-7525-2-68 (PMC539243; doi:10.1186/1477-7525-2-68)
Supplement: Additional File 1 — Appendix 1: Full details of the three palliative care outcome scales [file 1477-7525-2-68-S1.doc]

**Appendix 1: Full details of the three palliative care outcome scales**

# EuroQoL – 5

By placing a tick in one box in each group below, please indicate which statement best describes your own health state *today*?

1. **Mobility**

I have no problems in walking about

I have some problems in walking about

I am confined to bed

1. **Self-Care**

I have no problems with self-care

I have some problems washing or dressing myself

I am unable to wash or dress myself

1. **Usual Activities *(e.g. work, study, housework, leisure activities)***

I have no problems with performing my usual activities

I have some problems with performing my usual activities

I am unable to perform my usual activities

1. **Pain/ Discomfort**

I have no pain or discomfort

I have moderate pain or discomfort

I have extreme pain or discomfort

1. **Anxiety / Depression**

I am not anxious or depressed

I am moderately anxious or depressed

I am extremely anxious or depressed

**E6. General Health**

Patient evaluation in a 0-100 Visual Analogue Scale.

# Palliative Care Outcome Scale (POS)

Please answer the following questions circling the answer, which you think most accurately, describes how you have been feeling.

1. **Over the past *3 days*, have you been affected by pain?**

Not at all, no effect 0

Slightly - but not bothered to be rid of it 1

Moderately - pain limits some activity 2

Severely - activities or concentration markedly affected 3

Overwhelmingly - unable to think of anything else 4

1. **Over the past *3 days*, have other symptoms e.g. feeling sick, having a cough or constipation been affecting how you feel?**

No, not at all 0

Slightly 1

Moderately 2

Severely 3

Overwhelmingly 4

1. **Over the past *3 days*, have you been feeling anxious or worried about your illness or treatment?**

No, not at all 0

Occasionally 1

Sometimes - affects my concentration now and then 2

Most of the time - often affects my concentration 3

Can’t think of anything else - completely preoccupied 4

1. **Over the past *3 days*, have any of your family or friends been anxious or worried about you?**

No, not at all 0

Occasionally 1

Sometimes - it seems to affect their concentration 2

Most of the time 3

Yes, always preoccupied with worry about me 4

1. **Over the past *3 days*, how much information have you and your family or friends been given?**

Full information - always feel free to ask what I want 0

Information given but hard to understand 1

Information given on request but would have liked more 2

Very little given and some questions were avoided 3

None at all 4

1. **Over the past *3 days*, have you been able to share how you are feeling with your family or friends?**

Yes, as much as I wanted to 0

Most of the time 1

Sometimes 2

Occasionally 3

No, not at all with anyone 4

1. **Over the past *3 days*, have you felt that life was worthwhile?**

Yes, all the time 0

Most of the time 1

Sometimes 2

Occasionally 3

No, not at all 4

1. **Over the past *3 days*, have you felt good about yourself as a person?**

Yes, all the time 0

Most of the time 1

Sometimes 2

Occasionally 3

No, not at all 4

1. **Over the past *3 days*, how much time do you feel you have wasted on appointments relating to your healthcare, e.g. waiting around for transport or having the same tests repeated?**

None at all 0

Up to half a day wasted 1

More than half a day wasted 2

1. **Over the past *3 days*, have any practical matters resulting from your illness, either financial or personal, been addressed?**

Practical problems have been addressed and my affairs

are as up to date as I would wish 0

Practical problems are in the process of being addressed 1

Practical problems exist which were not addressed 2

I have had no practical problems 3

1. **If any, what have been your *main* problems in the last *three days*?**

1.

2.

# Herth Hope Index

Listed below are a number of statements. Read each statement and place a (X) in the box that describes how much you agree with that statement *right now*.

|  | | Strongly  Disagree | Disagree | Agree | Strongly  Agree |
| --- | --- | --- | --- | --- | --- |
|  | I have a positive outlook toward life |  |  |  |  |
|  | I have short, intermediate, and/ or long range goals |  |  |  |  |
|  | I feel all alone |  |  |  |  |
|  | I can see a light in a tunnel |  |  |  |  |
|  | I have faith that gives me comfort |  |  |  |  |
|  | I feel scared about my future |  |  |  |  |
|  | I can recall happy / joyful times |  |  |  |  |
|  | I have deep inner strength |  |  |  |  |
|  | I am able to give and receive caring / love |  |  |  |  |
|  | I have a sense of direction |  |  |  |  |
|  | I believe that each day has potential |  |  |  |  |
|  | I feel my life has value and worth |  |  |  |  |
