# Supplementary material for: Relationship between three palliative care outcome scales
Source: Health Qual Life Outcomes. 2004 Nov 29;2:68. doi: 10.1186/1477-7525-2-68 (PMC539243; doi:10.1186/1477-7525-2-68)
Supplement: Additional File 2 — Table 4: Summary Statistics and Principal Component Analysis (unrotated) of the three scales (POS-EQoL-Hope) in the historical sample. [file 1477-7525-2-68-S2.doc]

**Table 2: Summary Statistics and Principal Component Analysis (unrotated) of the three scales (POS-EQoL-Hope) in the historical sample.**

|  |  | **Summary statistics**  **(Combined Sample)** | | | | **PCA (unrotated) of**  **each individual scale** | | | | **PCA (unrotated) of the**  **three combined scales** | | | | |
| --- | --- | --- | --- | --- | --- | --- | --- | --- | --- | --- | --- | --- | --- | --- |
|  |  |  | | | | I | II | III | IV | I | II | III | IV | V |
| Scale | Item | **Mean** | SD | **Min** | **Max** |  |  |  |  | 25% | 35% | 43% | 49% | 54% |
| EQoL-1 | Mobility | 1.75 | .50 | 1 | 3 | **0.80** | *0.15* | *0.03* | *-* | -0.23 | **0.67** | -0.19 | -0.02 | 0.13 |
| EQoL-2 | Self-care | 1.48 | .61 | 1 | 3 | **0.77** | *0.29* | *-0.04* | *-* | -0.23 | **0.66** | -0.21 | 0.17 | 0.15 |
| EQoL-3 | Usual activities | 1.99 | .66 | 1 | 3 | **0.64** | *0.48* | *-0.11* | *-* | -0.08 | **0.71** | -0.13 | 0.31 | 0.28 |
| EQoL-4 | Pain-Discomfort | 1.76 | .59 | 1 | 3 | *0.44* | *-0.49* | **0.71** | *-* | -0.18 | 0.46 | 0.17 | **-0.64** | -0.14 |
| EQoL-5 | Anxiety-Depression | 1.48 | .57 | 1 | 3 | *0.33* | **-0.70** | ***-0.56*** | **-** | **-0.59** | 0.07 | **0.49** | 0.20 | -0.25 |
| EQoL-6 | Health Status | 61.9 | 19.6 | 0 | 100 | **-0.73** | *0.29* | *0.06* | *-* | **0.47** | **-0.51** | 0.11 | 0.09 | 0.17 |
| POS-1 | Pain Control | 1.41 | 1.00 | 0 | 3 | *0.39* | *0.24* | *-0.41* | **0.55** | -0.26 | 0.43 | 0.26 | **-0.58** | -0.10 |
| *POS-2* | Symptom Control | 1.10 | 1.00 | 0 | 4 | *0.32* | *-0.20* | *0.04* | **0.66** | -0.31 | 0.13 | -0.09 | -0.22 | 0.24 |
| POS-3 | Anxious/Worried | .8 | 1.02 | 0 | 4 | **0.66** | *0.08* | *-0.13* | *-0.31* | **-0.48** | 0.13 | **0.49** | 0.25 | -0.21 |
| *POS-4* | Family anxious | 1.56 | 1.32 | 0 | 4 | *0.43* | *0.21* | **-0.60** | *-0.03* | -0.16 | **0.46** | 0.32 | 0.12 | -0.16 |
| *POS-5* | Information | .09 | .42 | 0 | 3 | *0.25* | **0.73** | *0.37* | *-0.03* | -0.01 | 0.01 | **0.62** | -0.01 | **0.57** |
| POS-6 | Share feelings | .85 | 1.42 | 0 | 4 | ***0.53*** | *-0.23* | *0.48* | *-0.16* | **-0.54** | -0.24 | 0.05 | 0.09 | 0.19 |
| POS-7 | Life Worthwhile | .64 | .99 | 0 | 4 | **0.78** | *-0.20* | *0.23* | *-0.13* | **-0.69** | -0.07 | 0.10 | 0.23 | 0.07 |
| POS-8 | Feel Good | 1.09 | 1.28 | 0 | 4 | **0.76** | *-0.19* | *-0.05* | *0.00* | **-0.75** | 0.07 | 0.13 | 0.14 | -0.13 |
| *POS-9* | Time Wasted | .07 | .38 | 0 | 2 | *0.03* | **0.57** | *0.46* | *0.36* | 0.01 | -0.06 | 0.24 | -0.25 | **0.75** |
| *POS-10* | Practical matters | .43 | .90 | 0 | 4 | *0.08* | **0.58** | *-0.24* | *-0.38* | 0.10 | 0.13 | **0.52** | 0.34 | -0.03 |
| HOPE1 | Positive outlook | 3.34 | .70 | 1 | 4 | **0.54** | *0.08* | *-0.19* | *0.13* | **0.77** | 0.08 | 0.14 | 0.13 | -0.02 |
| *HOPE2* | Goals | 2.93 | .76 | 1 | 4 | *0.26* | **0.63** | *0.06* | *0.02* | **0.56** | -0.09 | 0.33 | -0.31 | -0.17 |
| HOPE3 | Alone | 3.24 | .93 | 1 | 4 | *0.04* | *-0.14* | **-0.72** | *0.21* | **0.57** | 0.23 | -0.32 | 0.16 | -0.10 |
| HOPE4 | Tunnel | 2.64 | .84 | 1 | 4 | *-0.10* | **0.88** | *0.01* | *0.08* | 0.40 | 0.01 | 0.22 | -0.40 | -0.17 |
| HOPE5 | Faith | 3.06 | .81 | 1 | 4 | *0.19* | *0.26* | *0.18* | **0.59** | **0.59** | 0.01 | 0.05 | 0.11 | 0.02 |
| HOPE6 | Scared of Future | 2.86 | .89 | 1 | 4 | *-0.01* | *0.03* | **-0.89** | *-0.19* | **0.49** | 0.18 | -0.40 | -0.10 | 0.11 |
| HOPE7 | Happy memories | 3.69 | .56 | 1 | 4 | *-0.21* | *0.07* | *-0.15* | **0.89** | **0.51** | 0.27 | 0.07 | 0.09 | 0.01 |
| *HOPE8* | Inner strength | 3.29 | .70 | 1 | 4 | **0.63** | *0.00* | *-0.09* | *0.16* | **0.71** | 0.21 | 0.06 | 0.11 | -0.16 |
| HOPE9 | Loving | 3.49 | .62 | 1 | 6 | 0.46 | *-0.31* | *0.07* | **0.52** | **0.51** | 0.33 | 0.09 | 0.16 | 0.08 |
| HOPE10 | Sense of direction | 3.06 | .75 | 1 | 4 | **0.84** | *0.10* | *-0.04* | *-0.13* | **0.75** | 0.06 | 0.21 | -0.05 | 0.04 |
| HOPE11 | Days have Potential | 3.27 | .64 | 1 | 4 | **0.97** | -0.10 | 0.01 | -0.17 | **0.69** | 0.19 | 0.20 | 0.15 | -0.05 |
| HOPE12 | Life has value | 3.30 | .73 | 1 | 4 | **0.87** | 0.06 | 0.10 | -0.01 | **0.77** | 0.02 | 0.23 | 0.08 | 0.07 |

* highest loading for each item is highlighted. If highest two differ in less than 0.10, both are highlighted.
